# Transient, self-limiting, antibody-negative myositis with venetoclax

**DOI:** 10.1016/j.clinsp.2024.100444

**Published:** 2024-07-10

**Authors:** Carla A. Scorza, Josef Finsterer, Fulvio A. Scorza

**Affiliations:** a Disciplina de Neurociência, Universidade Federal de São Paulo/Escola Paulista de Medicina (UNIFESP/EPM), São Paulo, SP, Brasil; b Neurology Neurophysiology Center, Vienna, Austria

*Dear Editor,*

Chronic Lymphatic Leukemia (CLL) can rarely be complicated by inclusion-body myositis.^1^^,^^2^ There are also two case reports about CLL with dermatomyositis.^3^^,^^4^ Transient, self-limiting myositis in CLL has not been reported.

The patient is a 69-year-old woman, height 172 cm, weight 78 kg, who was diagnosed with CLL in June 2006. In addition, her medical history was positive for diabetes, moderate renal insufficiency, polyneuropathy, arterial hypertension, hypothyroidism, sicca syndrome, and glaucoma surgery. Since Fluorescence In Situ Hybridisation (FISH) was negative in 2006, the wait-and-see strategy was initially pursued for CLL. However, in May 2007 progression occurred manifested by lymphadenopathy and splenomegaly, classified as RAI II and Binet B, for which she received five cycles of fludarabine and cyclophosphamide. Although re-staging in July 2012 showed a significant reduction in lymph node size, she was enrolled in the CLL-TRU.016 trial and received six cycles of bendamustin through August 2012. Despite this treatment, a relapse occurred in September 2014 with a lymphocyte doubling time < 6 months, platelet count < 100, and increased β2-microglobulin.

This time FISH revealed del 11q, and del 13q. Therefore, the patient received two cycles of bendamustin in October 2014 and one cycle of ofatumumab in December 2014. In March 2023 progressive thrombocytopenia and the BTKi mutation c.1442G>C were detected. However, the administration of ibrutinib was complicated by dermal side effects, which is why treatment was started using the MURANO regimen (Venetoclax plus Rituximab [RTX]). She received the last RTX treatment in January 2024. Almost four weeks later, the patient developed myalgias of the anterior thigh muscles. Clinical neurologic examination two days later revealed muscle soreness in the neck, decreased tendon reflexes in the upper and lower limbs, rapid lowering of the legs when attempting to hold the leg, and marked standing ataxia with a tendency to fall backward. Creatine-kinase was elevated to 2746 U/L (Table 1). A muscle MRI three days after the onset of myalgias revealed muscle edema of the left vastus intermedius muscle, the right vastus medialis muscle, and the right gluteus medialis muscle (Fig. 1). Non-specific myositis was diagnosed and in the following days myalgia and muscle enzymes resolved spontaneously (Table 1). The search for common causes of myositis remained negative. Myositis-specific antibodies and myositis-associated antibodies were negative as were antisynthetase antibodies and PCRs for viral infection. Current medications included empagliflozine, nebivolol, valsartane, levothyroxine, pregabalin, INN filgastrin, venetoclax, allopurinol, valacyclovir, and dexpanthenol.

**Table 1:** Blood chemical values before and during the myositis episode.

**Parameter**	RL	12.12.23	22.12.	10.1.24	18.1.24	7.2.24	8.2.24	9.2.24
**CK**	0‒145 U/L	ND	ND	ND	ND	2746	1450	1268
**GOT**	0‒34 U/L	19	ND	21	20	94	75	81
**GPT**	0‒34 U/L	11	ND	16	15	27	23	23
**LDH**	0‒246 U/L	234	ND	213	213	340	256	309
**LDH**	0‒246 U/L	234	ND	213	213	340	256	309
**Creatinine**	0.51‒0.95 mg/dL	1.18	1.27	1.04	1.27	1.42	1.32	1.2
**GFR**	90‒2001.7 m^2^KO	47	43	55	43	38	41	46
**Erythrocytes**	4.0‒50.0 T/L	3.4	3.8	3.5	3.9	3.9	3.6	3.5
**Leucocytes**	4.0‒10.0 G/L	1.8	1.9	10.4	7.2	3.2	1.3	1.2
**Thrombocytes**	150‒400 G/L	102	102	111	134	72	53	40
**CRP**	0.0‒4.9 mg/L	5.2	12.7	3.5	8.2	43.3	46.2	46.8

CK, Creatine-Kinase; CRP, C-Reactive Protein; GFR, Glomerular Filtration Rate; GOT, Glutamate Oxalate Transaminase; GPT, Glutamate Pyruvate Transaminase; LDH, Lactate Dehydrogenase; ND, Not Done, RL, Reference Limits.

**Fig. 1:**
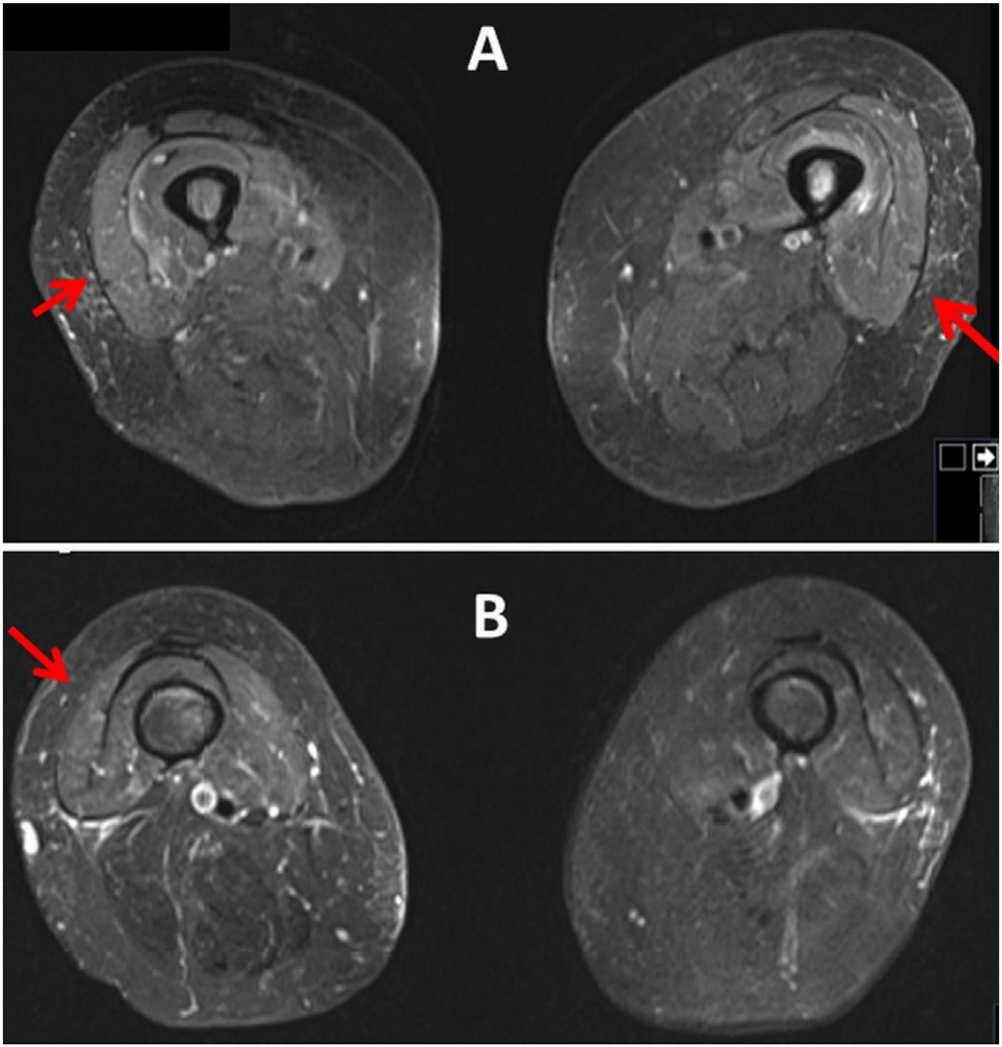
Muscle MRI (T2 STIR sequences) of the thigh and pelvic muscles three days after admission showing edema of the left intermedial vastus muscle, of the right medial vastus muscle, and right medial gluteus muscle arrows).

The patient presented is of interest because of myositis of the thigh and pelvic muscles during chemotherapy with venetoclax plus RTX for CLL. Possible causes of myositis in the index patient were infectious disease, autoimmune disease, paraneoplasia, or drug-associated diseases. Arguments against infection are that the patient had no fever before the onset of myalgia, that CRP was almost normal 18 days before the onset of myalgia, that the PCR was negative for CMV, EBV, influenza, and SARS-CoV-2, and that leukocyte count did not increase but decreased since the onset of myalgia. The fact that ANA, ANCA were negative and that myositis-associated antibodies and myositis-specific antibodies were negative speaks against an immunological cause. Another argument against autoimmune myositis is that she did not develop manifestations such as dysphagia, calcinosis, rheumatoid arthritis, lupus, or scleroderma. Furthermore, no subtypes of Idiopathic Inflammatory Myositis (IIM) could be identified. Dermatomyositis was excluded because the patient did not have typical cutaneous features (heliotrope rash, Gottron papules, shawl sign). Inclusion body myositis was excluded because there was no progression but spontaneous regression and IBM occurs more frequently in men than females. Polymyositis could not be definitively excluded because the patient did not undergo muscle biopsy to detect abnormal activation of CD8 T-lymphocytes and extrafusal expression of major histocompatibility complex-I.^5^ Necrotizing myositis was excluded due to the absence of SRP and HMGCR antibodies and only mild CK elevation. However, an argument in favor of paraneoplastic IIM is that polymyositis, dermatomyositis, and inclusion body myositis have already been reported in association with CLL.^1^^,^^3^^,^^6^ Whether venetoclax played a pathophysiological role remains speculative. Although no cases with venetoclax-associated myositis or rhabdomyolysis have been reported to date, it cannot be definitively excluded that it played a pathophysiological role. Filgastrin was excluded as a cause of myositis because there are no reports of such side effects in the literature. The long latency period between the last use of RTX and the onset of myalgias suggests that there was no causal relationship between RTX and the onset of CK-elevation. Whether the elevated CRP reflects infectious disease, CLL, or simply myositis remains speculative. Since common infectious diseases have been excluded and CLL is not usually associated with elevated CRP, it most likely reflects myositis.

It is concluded that transient, self-limiting myositis may occur in patients with CLL receiving venetoclax. Although not previously reported, a causal relationship between the two cannot be definitively ruled out.

## Statement of ethics

1. The study was approved by the institutional review board (responsible: Finsterer J.) on the 4^th^ of November 2022.
2. Written informed consent was obtained from the patient for publication of the details of their medical care and any accompanying images.

Data availability statement

Data that support the findings of the study are available from the corresponding author.

## Compliance with ethics guidelines

This article is based on previously conducted studies and does not contain any new studies with human participants or animals performed by any of the authors.

## Authors’ contributions

JF: Design, literature search, discussion, first draft, critical comments, final approval.

## Funding

No funding was received.

## Conflicts of interest

The author declares that the research was conducted in the absence of any commercial or financial relationships that could be construed as a potential conflict of interest.

## References

[1] Beck EH, Amato AA, Greenberg SA. (2014). Inclusion body myositis and chronic lymphocytic leukemia: a case series. Neurology.

[2] Arnardottir S, Ansved T, Nennesmo I, Borg K. (2001). Report of a patient with inclusion body myositis and CD8+ chronic lymphocytic leukaemia-post-mortem analysis of muscle and brain. Acta Neurol Scand.

[3] Kumar S, Gogia A, Gupta R, Mallick S. (2020). Chronic Lymphocytic Leukemia with dermatomyositis: a therapeutic challenge. Turk J Haematol.

[4] Ishida T, Aikawa K, Tamura T, Yoshida K, Mikuni C, Fujita M (1995). Chronic lymphocytic leukemia associated with nephrotic syndrome and dermatomyositis. Intern Med.

[5] Sarwar A, Dydyk AM, Jatwani S. Polymyositis. [Updated 2023 Feb 7]. In: StatPearls [Internet]. Treasure Island (FL): StatPearls Publishing; 2024 Jan. Available from: https://www.ncbi.nlm.nih.gov/books/NBK563129/. 33085276

[6] Cherin P, Piette JC, Herson S. (1994). Polymyosite et leucémie lymphoïde chronique [Polymyositis and chronic lymphoid leukemia]. Rev Med Interne.

